# Thymic Hyperplasia Mimicking Lymphoma in a Pediatric Patient: A Case Report

**DOI:** 10.7759/cureus.88620

**Published:** 2025-07-23

**Authors:** Khadija Belcadi Abassi, Azzeddine Laaraje, Radi Abdelilah, Rachid Abilkassem

**Affiliations:** 1 Pediatrics, Mohammed V Military Hospital, Rabat, MAR

**Keywords:** child, differential diagnosis, mediastinal mass, radiology, thymic hyperplasia

## Abstract

Mediastinal masses in children often raise concern for malignant conditions, particularly lymphoma. The thymus, which is normally prominent in infants and young children, begins its physiological involution around the age of six to seven years; however, this process is gradual and can extend into adolescence. Thymic hyperplasia beyond this age is uncommon, and it can mimic a mediastinal tumor on imaging. We report a case of benign thymic hyperplasia in a 10-year-old asymptomatic patient who was initially suspected of having lymphoma based on an anterior-superior mediastinal mass seen on an external CT scan. Imaging showed a homogeneous mass occupying the thymic lodge, associated with bilateral cervical lymphadenopathy. Although the clinical and imaging findings were suggestive of benign thymic hyperplasia, the presence of a large anterior mediastinal mass with bilateral cervical lymphadenopathy warranted exclusion of T-lymphoblastic lymphoma. Therefore, a mediastinal biopsy was performed, confirming the benign nature of the lesion. This report underscores the importance of considering malignancy in the differential diagnosis and using biopsy judiciously to achieve diagnostic certainty when imaging alone cannot fully exclude aggressive pathology.

## Introduction

Mediastinal masses in children are always a cause for concern due to the associated high frequency of malignant etiologies, particularly lymphomas. The thymus, a lymphoid organ located in the anterior mediastinum, plays a key role in the development and maturation of T-lymphocytes for immune function. While it is typically prominent in infants and young children, it physiologically involutes after the age of six to seven years [1]. Thus, thymic hyperplasia, defined as an enlargement of the thymus without abnormal histological changes, is uncommon beyond this age and can be mistaken for a mediastinal tumor. This diagnostic confusion may lead to unnecessary invasive procedures and considerable anxiety for families and caregivers. It is therefore important to recognize benign forms of thymic hyperplasia, even in atypical age groups, and to carefully correlate clinical, radiological, and laboratory findings before concluding malignancy [2]. We report the case of a 10-year-old patient initially suspected of having mediastinal lymphoma but who was eventually diagnosed with true thymic hyperplasia (TTH).

## Case presentation

**Figure 1:**
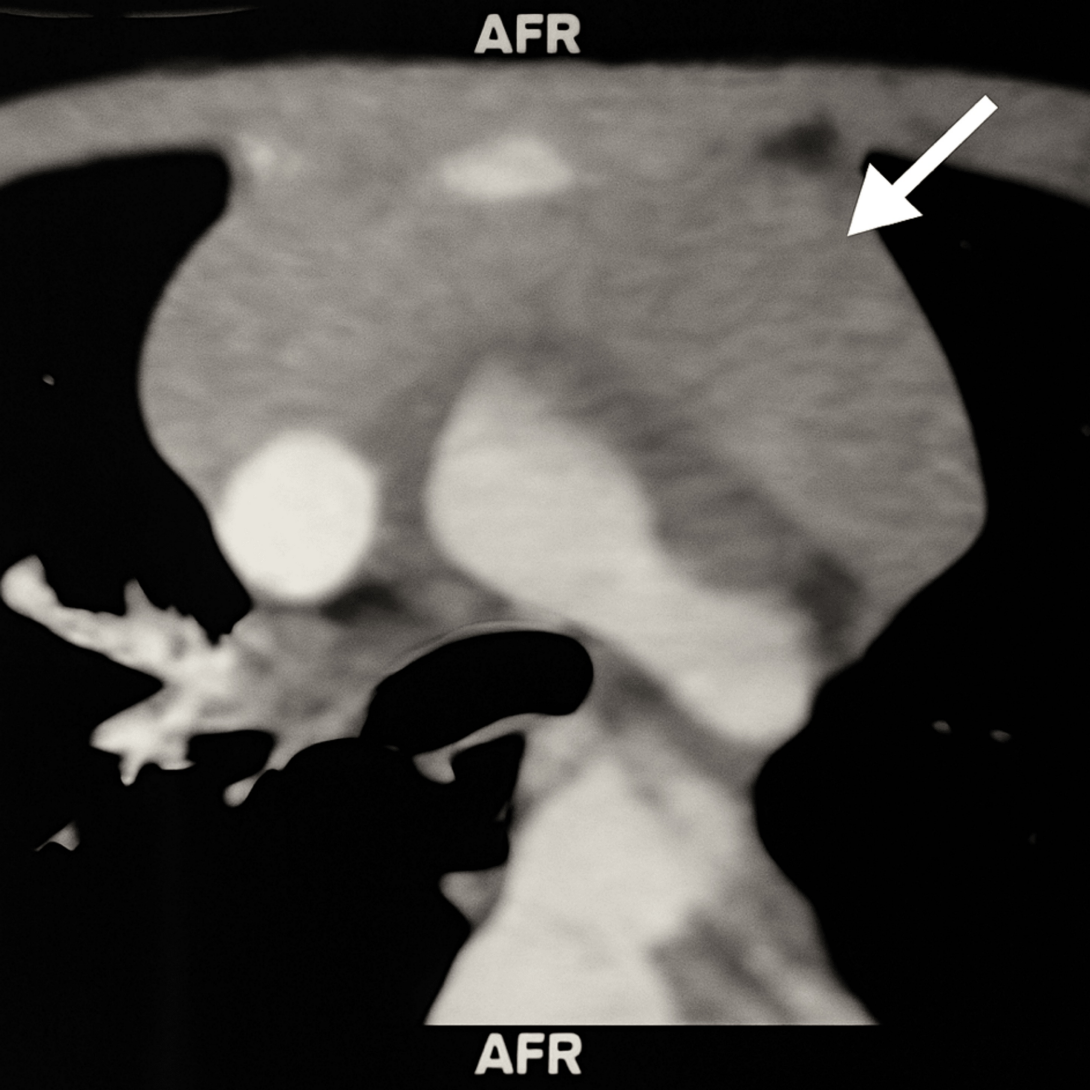
Chest CT scan showing an anterior-superior mediastinal soft tissue mass measuring 74 × 24 mm (white arrow) CT: computed tomography

**Figure 2:**
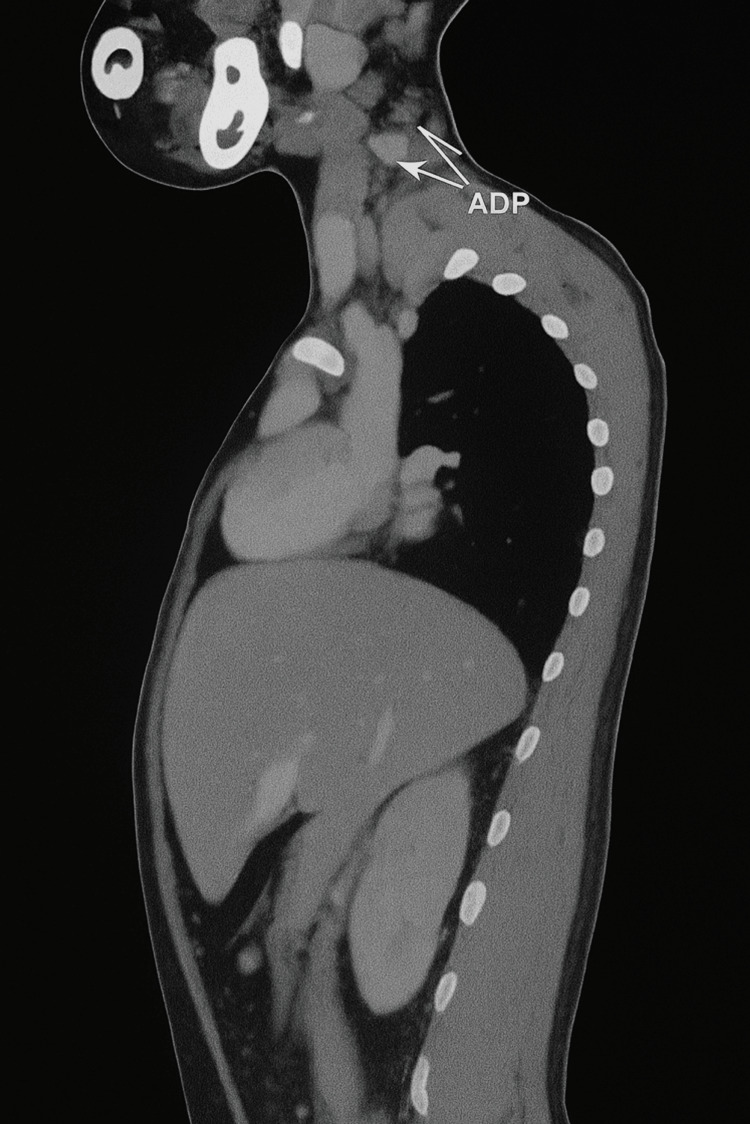
Bilateral cervical lymphadenopathy (white arrows)

The patient was initially referred to our center for further evaluation of a mediastinal mass incidentally discovered on a chest X-ray performed for persistent cough. The imaging revealed an anterior-superior mediastinal tissue process occupying the thymic lodge, with a homogeneous, pseudotumoral appearance measuring 74 × 24 mm, molding vascular and tracheobronchial structures without invading them (Figure 1). Bilateral cervical lymphadenopathy was also noted, with the largest measuring 2 × 1 cm (Figure 2). These findings raised suspicion of a tumor, primarily lymphoma, but also thymoma or a germ cell tumor, prompting a transfer to our facility.

On physical examination, the patient was in good general health, with no signs of superior vena cava syndrome and no respiratory distress. Cardiorespiratory, abdominal, and neurological exams were unremarkable. Laboratory tests revealed normal values across all parameters; the complete blood count (CBC) showed a white blood cell count of 7,200/mm³ (reference range: 4,000-10,000/mm³), hemoglobin level of 13.2 g/dL (reference: 11.5-15.5 g/dL), and platelet count of 280,000/mm³ (reference: 150,000-400,000/mm³). Inflammatory markers were within normal limits, with C-reactive protein (CRP) at 2 mg/L (reference: <5 mg/L) and erythrocyte sedimentation rate (ESR) at 10 mm/h (reference: <20 mm/h). Lactate dehydrogenase (LDH) was measured at 180 U/L (reference: 135-225 U/L). Tumor markers, including alpha-fetoprotein (AFP) at 3 ng/mL (reference: <10 ng/mL) and beta-human chorionic gonadotropin (β-HCG) at <1 IU/L (reference: <5 IU/L), were also within normal ranges. These findings supported the absence of any malignant or inflammatory process.

**Figure 3:**
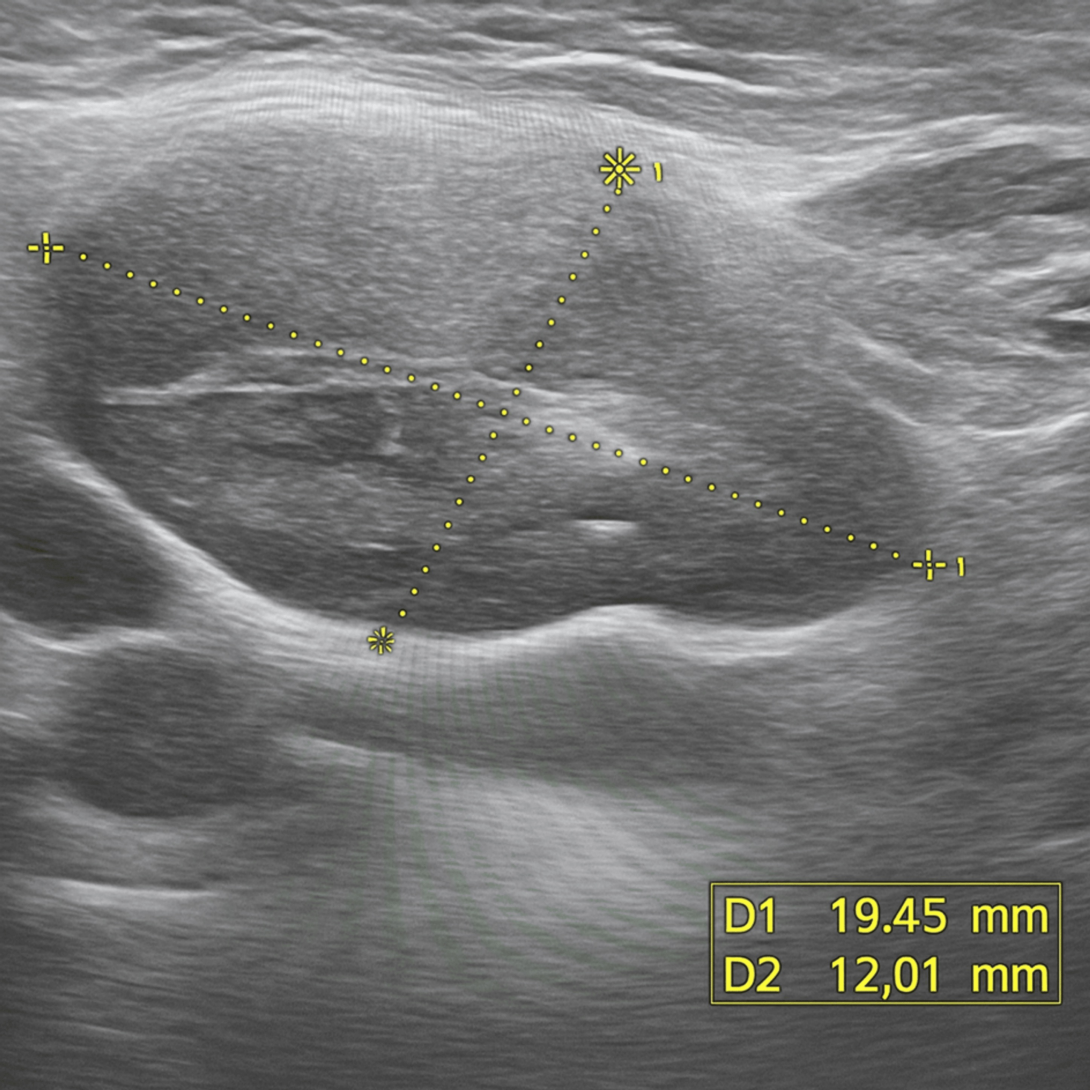
Ultrasound image of the largest cervical lymph node, showing features suggestive of benignity

A cervical ultrasound performed at our center re-evaluated the initial interpretation. It showed a homogeneous, isoechoic mass whose echotexture was reminiscent of the thymus. The associated cervical lymph nodes had an oval shape with preserved central echogenic hilum, regular borders, central vascularization on Doppler, and no signs of necrosis, calcification, or capsular disruption-features consistent with benign lymphadenopathy (Figure 3). A CT scan review confirmed homogeneous thymic hyperplasia with no suspicious findings. To rule out any doubt, a mediastinal biopsy was performed and was completely normal on histological examination, showing no tumor proliferation.

Given the absence of symptoms and the histologically confirmed benign nature, a conservative management approach was adopted. The patient was placed under regular monitoring, which included clinical examinations and follow-up cervical ultrasounds. The ultrasound evaluations showed a gradual decrease in the size of the enlarged thymus, with complete resolution of the associated cervical lymphadenopathy. The patient remained asymptomatic throughout, and no new signs suggestive of malignancy appeared. This favorable evolution confirmed the diagnosis of TTH and validated the conservative approach, avoiding unnecessary interventions.

## Discussion

The thymus is a primary lymphoid organ located in the anterior mediastinum, embryologically derived from the third and fourth pharyngeal pouches [3]. It plays a key role in T-cell maturation during fetal life and early childhood [4]. Thymic hyperplasia is rarely reported beyond the age of six to seven years, the period during which the thymus begins its physiological involution, progressively replaced by adipose tissue [1]. Nevertheless, persistent thymic hyperplasia has been reported in older children, though it remains exceptional [5]. There are two classic forms of thymic hyperplasia: TTH, defined as an increase in size without histological architectural changes, and lymphofollicular hyperplasia, usually associated with autoimmune diseases [3].

TTH is characterized by enlargement of the thymus with preservation of its histological architecture and immunological components. It differs from lymphofollicular hyperplasia, often linked to autoimmune diseases such as myasthenia gravis [3,6,7]. Although TTH is considered idiopathic in many cases, several possible associations have been described in the literature. Post-stress rebound hyperplasia may occur following recovery from severe infections, chemotherapy, steroid therapy, or radiation, where the thymus re-expands rapidly as part of immune system recovery [5] Graves’ disease, although rare in children, has been reported as a cause of thymic enlargement due to thyrotropin receptor stimulation, and this enlargement is often reversible with the treatment of hyperthyroidism [7]. In addition, children recovering from serious illnesses such as burns, trauma, or malnutrition may exhibit thymic rebound hypertrophy during the recovery phase.

Finally, in some patients, no identifiable cause is found despite thorough evaluation, necessitating careful exclusion of other anterior mediastinal masses such as lymphomas or germ cell tumors, and its occurrence beyond the age of six to seven years is considered unusual [1,5]. Clinically, thymic hyperplasia is usually asymptomatic and incidentally discovered on imaging [3]. However, in some cases, it may present with mediastinal compression symptoms such as dyspnea, cough, dysphagia, or recurrent respiratory infections [4]. The main differential diagnosis for an anterior mediastinal mass in children remains lymphoma, particularly lymphoblastic lymphoma, but germ cell tumors or thymomas should also be considered. On imaging, TTH typically presents with characteristic CT features as described by Priola et al. [4]: an anterior mediastinal lesion with a triangular bipyramidal shape, symmetrical like a normal thymus, homogeneous in density, and often containing microscopic fat. MRI, using phase contrast sequences, can also identify this microscopic fat infiltration, a feature not seen in tumors [8]. Cervical ultrasound may show a well-defined, isoechoic mass compared to the liver, preserving thymic architecture [9].

In our case, the finding of a homogeneous anterior-superior mediastinal mass with bilateral cervical lymphadenopathy initially suggested lymphoma. However, the patient was completely asymptomatic, with no general or specific clinical signs. Physical examination was reassuring, with no superior vena cava syndrome or respiratory distress, and all laboratory findings (CBC, CRP, LDH, tumor markers) were normal. Cervical ultrasound played a pivotal role in the diagnostic approach. It revealed a homogeneous, isoechoic mass consistent with typical thymic echotexture, along with benign-appearing lymph nodes (oval shape, visible hilum, regular contours, central Doppler vascularization, and absence of necrosis or calcification) [6]. A thoracic CT scan confirmed the thymic appearance without invasive features. Histological examination of the mediastinal biopsy confirmed the benign nature of the lesion, showing preserved thymic architecture with normal corticomedullary differentiation, abundant lymphocyte populations, and scattered Hassall’s corpuscles, without evidence of cellular atypia, lymphoid proliferation, or malignancy.

Although PET-CT could have added diagnostic value (since lymphomas typically show high FDG uptake while TTH usually demonstrates absent or minimal uptake), it was not performed in this case because CT and ultrasound findings, together with histological confirmation, were sufficient for diagnosis. Moreover, in Morocco, access to PET-CT is limited due to its high cost and is generally reserved for more complex or equivocal cases. Nonetheless, its potential utility in evaluating mediastinal masses is acknowledged.

This case fits within the rare context of TTH diagnosed beyond early childhood. When the thymus reaches a disproportionately large size relative to age (exceeding the cardiac silhouette on X-ray or representing more than 2% of body weight), it is classified as massive thymic hyperplasia (MTH) [1,4,5]. About 50 cases of MTH have been reported in pediatric literature [4,10,11], mostly between 1 and 15 years old, with a male predominance and often symptomatic presentation (cough, dyspnea, respiratory infections) [12]. Management of MTH is not standardized. Corticosteroid therapy has been attempted in some cases to reduce thymic size, but outcomes are often disappointing, with either no response or rebound hypertrophy after discontinuation [13-15]. Most reported cases were treated surgically, particularly when the mass was large or causing compressive symptoms [10,12].

This report highlights the importance of considering TTH, even at an older age, in the differential diagnosis of mediastinal masses in children. A multidisciplinary approach correlating clinical, biological, and radiological findings is essential to avoid unnecessary invasive procedures or inappropriate treatments. In our patient, the conservative approach was made possible thanks to a thorough analysis combining clinical, biological, ultrasound, and CT data. The absence of symptoms, radiological stability, and histological confirmation of benignity led to the decision to pursue careful monitoring rather than intervention.

## Conclusions

This report illustrates the diagnostic challenge posed by TTH beyond the usual involution age. Its pseudotumoral appearance on imaging, combined with cervical lymphadenopathy, can easily mimic lymphoma and lead to unnecessary invasive investigations. Careful consideration of the child’s clinical status, absence of systemic symptoms, reassuring laboratory workup, and detailed imaging analysis (especially cervical ultrasound) are crucial for a rational diagnostic approach. This report highlights the importance of a multidisciplinary strategy to avoid misdiagnosis and ensure appropriate management. While non-invasive methods can strongly suggest a benign process, this experience also underlines that histological confirmation may still be necessary in cases where diagnostic uncertainty persists, to provide definitive reassurance and guide management appropriately.
